# Impact of a functional polymorphism in the PAR-1 gene promoter in COPD and COPD exacerbations

**DOI:** 10.1152/ajplung.00128.2014

**Published:** 2014-06-27

**Authors:** Manuela Platé, Phillippa J. Lawson, Michael R. Hill, Jennifer K. Quint, Meena Kumari, Geoffrey J. Laurent, Jadwiga A. Wedzicha, Rachel C. Chambers, John R. Hurst

**Affiliations:** ^1^Centre for Inflammation and Tissue Repair, University College London, London, United Kingdom;; ^2^Centre for Respiratory Medicine, University College London, London, United Kingdom; and; ^3^Faculty of Population Health Sciences, University College London, London, United Kingdom

**Keywords:** PAR-1, *F2R*, SNP, COPD, COPD exacerbation

## Abstract

Proteinase-activated receptor-1 (PAR-1) plays a key role in mediating the interplay between coagulation and inflammation in response to injury. The aim of this study was to investigate the role of the promoter single-nucleotide polymorphism (SNP) rs2227744G>A in modulating PAR-1/*F2R* gene expression in the context of chronic obstructive pulmonary disease (COPD) and COPD exacerbations. The function of the rs2227744G>A SNP was investigated by using reporter gene assays. The frequency of the polymorphism in the UK population was assessed by genotyping 8,579 healthy individuals from the Whitehall II and English Longitudinal Study of Ageing cohorts. The rs2227744G>A SNP was genotyped in a carefully phenotyped cohort of 203 COPD cases and matched controls. The results were further replicated in two different COPD cohorts. The minor allele of the rs2227744G>A polymorphism was found to increase *F2R* expression by 2.6-fold (*P* < 0.001). The rs2227744G>A SNP was not significantly associated with COPD, or with lung function, in all cohorts. The minor allele of the SNP was found to be associated with protection from frequent exacerbations (*P* = 0.04) in the cohort of COPD patients for which exacerbation frequency was available. Considering exacerbations as a continuous variable, the presence of the minor allele was associated with a significantly lower COPD exacerbation rate (3.03 vs. 1.98 exacerbations/year, Mann-Whitney *U*-test *P* = 0.04). Taken together, these data do not support a role for the rs2227744G>A *F2R* polymorphism in the development of COPD but suggest a protective role for this polymorphism from frequent exacerbations. Studies in separate cohorts to replicate these findings are warranted.

inflammation of the lung parenchyma and airways is a central mechanism in the pathogenesis of obstructive lung diseases. The nature, magnitude, and pathways responsible for the initiation and persistence of inflammation vary between diseases, but upstream signaling responses may be activated ubiquitously. One such generic pathway includes the activation of the seven transmembrane domain G protein-coupled receptor proteinase-activated receptor-1 (PAR-1) in response to the local activation of the coagulation cascade (27). PAR-1 acts as cellular sensor of tissue injury and plays a pivotal role in orchestrating inflammatory and fibroproliferative responses during both normal wound healing, and a range of pathological contexts across all major organ systems (25). The major activators of PAR-1 include key proteinases of the coagulation cascade such as thrombin and factor Xa, the latter either alone or as part of the more potent tissue factor-factor VII-factor Xa ternary complex (6), although it is increasingly recognized that PAR-1 can also be activated by noncoagulation proteinases, including MMP-1 (5).

Chronic obstructive pulmonary disease (COPD) is characterized by progressive airflow obstruction that is only partly reversible, inflammation in the airways, and systemic effects or comorbidities. The main cause in the developed world is smoking tobacco in genetically susceptible individuals, but other factors have also been identified (9). Approximately 1% of COPD is associated with functional α_1_-antitrypsin deficiency (36), which provides the best model of genetic susceptibility to COPD. Current evidence, including data obtained in genomewide association studies (GWAS), shows that susceptibility is complex and may relate in part to epigenetic phenomena (33). COPD is a heterogeneous condition comprising many important clinical phenotypes that may have a different natural history or treatment response. Illustrating the potential importance of PAR-1 signaling in COPD, we have previously shown that PAR-1 knockout mice develop emphysema (3) but are protected from chronic bronchitis, supporting the notion that PAR-1 signaling pathways may influence clinical phenotype in this disease context. Episodes of acute functional deterioration, known as exacerbations, punctuate the natural history of COPD and account for most of the morbidity, mortality, and health care costs associated with this condition (12). There are several potential triggers, and exacerbations have been associated with airway infection, exposure to pollutants, or stimuli that affect expiratory airflow limitation (19). Some patients appear intrinsically more susceptible to exacerbations than others (18), and an important phenotype in COPD is the “frequent exacerbator” (35). The role of PAR-1 in this group of patients has not been previously examined, but COPD is associated with a hypercoaguable state (2, 20, 39). Moreover, an association has recently been demonstrated between thrombocytosis and significantly increased 1-year mortality following admission with acute COPD exacerbation, and PAR-1 represents the major thrombin receptor on human platelets (17). Interestingly, the common polymorphism rs2227744G>A in *F2R* (PAR-1 coding gene) promoter has been associated with inflammation and myocardial infarction (MI) in two Swedish cohorts (15). We now hypothesize that PAR-1 signaling influences susceptibility to COPD and the development of specific clinically relevant phenotypes, such as the frequent exacerbator. To address this hypothesis, we assessed the impact of the rs2227744G>A variant on *F2R* promoter activity using reporter gene assay experiments. We then assessed the polymorphism frequency in the general UK population, genotyping 8,579 healthy individuals from the English Longitudinal Study of Ageing (ELSA) and Whitehall II cohorts. Finally, we explored whether this PAR-1 polymorphism predisposes to susceptibility to COPD and/or frequent COPD exacerbations.

## MATERIALS AND METHODS

### Polymorphism Characterization

#### Plasmid preparation.

A 5.2-kb *F2R* promoter was excised from a HBAC clone (CTD-2384B11, Life Technologies, Carlsbad, CA) and inserted into pGL3 Basic Vector (Promega, Madison, WI) to produce the pGL3-F2R-wt plasmid. The presence and accuracy of the insert was confirmed by restriction mapping and sequencing by using the ABI PRISM BigDye Terminator v3.1 Cycle sequencing kit (Applied Biosystems, Foster City, CA), following the manufacturer's instructions. The rs2227744A allele was introduced in the wild-type plasmid by site-directed mutagenesis by using the QuickChange II XL Site-Directed Mutagenesis Kit (Stratagene, La Jolla, CA) as indicated by the manufacturer, producing the pGL3-F2R-rs227744A plasmid. Large-scale recovery of plasmid DNA to use for transient transfections was performed by using the EndoFree Plasmid MAXI Kit (QIAGEN, Hilden, Germany), according to the manufacturer's instructions.

#### Cell culture and transfections.

HeLa (human cervix carcinoma cell line) cells were grown at 37°C in a humidified atmosphere of 5% CO_2_ and 95% air in Dulbecco's modified Eagle's medium (DMEM, Life Technologies) supplemented with 10% fetal bovine serum (Life Technologies), 2 mM glutamine (Life Technologies), and antibiotics (100 IU/ml penicillin and 100 μg/ml streptomycin, Life Technologies). When 50% confluent, cells were transfected with 1 μg of the wild-type plasmid, the mutant plasmid, or the empty pGL3 vector plus 0.05 μg pRL-TK *Renilla* vector as an internal control to correct for differences in transfection efficiency and cell viability. The transfection was accomplished with the LID vector transfection system as described (1).

#### Firefly luciferase and Renilla luciferase assays.

Transcriptional activity was assayed by measuring luciferase activity. The activities of firefly and *Renilla* luciferase were measured 18 h after transfection by using the Dual-Luciferase Reporter Assay System (Promega). All transfections were performed in seven replicates and each construct was tested in three independent experiments using two different batches of plasmid preparation. The luciferase activity was measured with the TROPIX TR717 Microplate Luminometer (PE Applied Biosystems). For each construct, the values of firefly luciferase activity in different experiments were normalized against the corresponding values of *Renilla* luciferase (all expressed as relative luminescent units). Normalization was performed for each sample before comparing test groups.

### Subjects

#### Control subjects.

A total of 8,579 control subjects were studied in the Whitehall II and ELSA cohorts, which have been previously described (23, 38). Individuals with lung disease were excluded from the control cohorts for the purpose of this study. Among these, 609 age-, sex-, and smoke-matched controls were selected randomly for the case/control association analyses with the COPD cohort. Current and former smokers were aggregated in the single category of smokers. The remaining 1,133 Whitehall II and 1,094 ELSA smoker controls were included in the case/control association analyses with the Whitehall II and ELSA COPD cohorts, respectively.

#### Patients.

Two hundred and three COPD patients from the London COPD cohort were recruited and monitored as previously described (11). COPD was defined by a postbronchodilator forced expiratory volume in 1 s (FEV_1_) of ≤80% predicted, FEV_1_/forced vital capacity (FVC) <0.7, and β_2_-agonist reversibility on FEV_1_ of <15% and/or 200 ml. Patients were excluded if they had a history of other significant respiratory diseases, for example asthma. The patients completed daily diary cards recording any increase in daily respiratory symptoms (11), allowing us to prospectively and accurately record their exacerbations as previously described (32). The diary card definition of exacerbation has been validated against changes in quality of life (35), inflammatory markers (4), and FEV_1_ decline (11). This allowed us to classify patients as frequent (≥3 exacerbations in the previous year) and infrequent (<3 exacerbations in the previous year) exacerbators. Exacerbation definitions based on symptom criteria capture all events and therefore a frequent exacerbator is defined as ≥3 exacerbations in the previous year, rather than ≥2, which would apply in studies using a health-care utilization approach.

COPD patients in the Whitehall II (318) and ELSA (364) cohorts were defined at baseline as those who had smoked, who did not report a diagnosis of asthma, and had both FEV_1_ <80% and FEV_1_/FVC <0.7.

This study was conducted in accordance with the amended Declaration of Helsinki. Local institutional review boards or independent ethics committees approved the protocol, and written, informed consent was obtained from all patients. London COPD cohort: Royal Free Hospital; REC reference number 05/Q0501/126. ELSA: IRB00002308; MREC reference number MREC/04/006. Whitehall II: Joint University College London/University College London Hospital Committees on the Ethics of Human Research (Committee Alpha).

### Polymorphism Genotyping

Genotyping of the Whitehall II and ELSA cohorts was performed by KBiosciences by using the KASPar assay (LGC, Middlesex, UK), based on the Kompetitive Allele Specific PCR (KASP) technology. Genotyping error rates were examined from a repeat of 10% of samples in ELSA and 5% of samples in Whitehall II.

Within the COPD study, the rs2227744G>A single-nucleotide polymorphism (SNP) was genotyped by TaqMan assay (Applied Biosystems). In the COPD cohort random duplicated testing of 5% of the samples was performed and 10% of the samples genotypes were confirmed by direct sequencing.

In all cases the error rate was found to be <1%.

### Statistical Analyses

In reporter gene assay experiments, differences between plasmids were examined by univariate analysis. Reproducibility across repeat experiments was investigated by including the replicate experiments as a fixed factor.

For phenotypic data, categorical data were tested for difference between cases and controls by means of a χ^2^ test and continuous data were tested by the two-tailed Student *t*-test.

For genotypic data, all procedures were performed with the software package PLINK v1.07 (31). Hardy-Weinberg equilibrium was tested by the exact test described by Wigginton et al. (41) and implemented in the PLINK software. For comparison between cases with age-, sex-, and smoke-matched controls, a standard case-control analysis using allelic χ^2^ test to provide asymptotic *P* values, odds ratios, and 95% confidence intervals for the minor allele was used. For comparison between frequent and infrequent exacerbators, given the small size of the cohort, a Fisher's exact test was used (13). Logistic regression with correction for age and sex was used for the Whitehall II and ELSA COPD cohorts. For lung function analyses a Wald test was used. Values of *P* < 0.05 were considered to indicate statistical significance. The Mann-Whitney *U*-test was used to examine the relationship between allele frequencies and number of exacerbations.

## RESULTS

### F2R Haplotypes and Promoter Activity

To determine whether the SNP rs2227744G>A is functional, the rs2227744A minor allele was inserted into a plasmid containing the PAR-1 promoter upstream of the luciferase gene and the promoter activity was assessed in transient transfection experiments using HeLa cells. These studies revealed that the presence of the minor allele A of the SNP rs2227744G>A confers 2.6 ± 0.31-fold higher basal activity compared with the wild-type allele (*P* < 0.001), with no further increase seen following stimulation with TNF (10 ng/ml; data not shown).

### Phenotypic and Genotypic Characteristics of the Control UK Cohort

The frequency of the rs2227744G>A SNP was assessed in 8,579 individuals with no reported respiratory disease, who participated in the ELSA or Whitehall II study, as a representative sample of the UK Caucasian healthy population. The frequency of the minor allele was found to be 0.45. The SNP was in Hardy-Weinberg equilibrium.

### Phenotypic and Genotypic Characteristics of the COPD Cohort

The characteristics of the COPD patients and age-, sex-, and smoke-matched healthy control subjects selected from ELSA and Whitehall II are reported in Table 1 and demonstrate that the groups were well matched and that the COPD patients had obstructive lung function typical of that condition. The characteristics of the ELSA and Whitehall II COPD cases and controls are summarized in Table 2.

**Table 1.** Clinical characteristics of the COPD patients and matched healthy controls from ELSA and Whitehall II

	COPD (*n* = 203)				Controls (*n* = 609)	
Characteristics	IE (*n* = 136)	FE (*n* = 67)	*P* Value IE vs. FE	All	All	*P* Value COPD vs. Control
Age, yr	71.4 ± 8.9	70.7 ± 8.7	0.61	71.2 ± 8.8	71.2 ± 8.8	
Male	84 (62%)	36 (54%)	0.05	120 (59%)	360 (59%)	
Ever smoked, %	100	100		100	100	
FEV_1_, liters	1.22 ± 0.54	1.13 ± 0.43	0.17	1.19 ± 0.50	2.24 ± 0.72	<0.001
FEV_1_, % predicted	50 ± 20	48 ± 17	0.49	49 ± 19	92 ± 21	<0.001
FVC, liters	2.58 ± 0.97	2.39 ± 0.90	0.17	2.52 ± 0.95	2.97 ± 0.99	<0.001
FEV_1_/FVC	0.48 ± 0.14	0.49 ± 0.15	0.76	0.48 ± 0.14	0.76+±0.08	<0.001

Data are presented as means ± SD or number with % in parentheses. COPD, chronic obstructive pulmonary disease; FEV_1_, forced expiratory volume in 1 s; FVC, forced vital capacity; IE, infrequent exacerbators; FE, frequent exacerbators.

**Table 2.** Clinical characteristics of the COPD patients and healthy controls from ELSA and Whitehall II

	WHII			ELSA		
Characteristics	Cases (*n* = 318)	Controls (*n* = 1133)	*P* Value	Cases (*n* = 364)	Controls (*n* = 1094)	*P* Value
Age, yr	45.73 ± 6.1	43.43 ± 5.7	<0.001	71.40 ± 9.6	66.03 ± 8.44	<0.001
Male	199 (63%)	860 (76%)	<0.001	207 (57%)	510 (47%)	<0.001
Ever smoked, %	100	100		100	100	
FEV_1_, liters	2.04 ± 0.68	3.05 ± 0.67	<0.001	1.38 ± 0.60	2.46 ± 0.77	<0.001
FEV_1_, % predicted	59.16 ± 14.83	84.52 ± 11.79	<0.001	56.16 ± 18.56	99.39 ± 43.43	<0.001
FVC, liters	3.49 ± 0.88	3.96 ± 0.77	<0.001	2.62 ± 0.97	3.20 ± 1.06	<0.001
FEV_1_/FVC	0.58 ± 0.11	0.77 ± 0.06	<0.001	0.53 ± 0.14	0.78 ± 0.08	<0.001

Data are presented as means ± SD or number with % in parentheses. WHII, Whitehall II.

The frequency of the rs2227744G>A polymorphism was assessed in the control and COPD subjects. Genotype frequencies were analyzed to verify whether their distribution fit expectations under the Hardy-Weinberg equilibrium. This SNP did not show a statistically significant deviation from the equilibrium in cases or controls (or both) (data not shown). Allelic χ^2^ analyses on cases and controls revealed that the SNP was not associated with the development of COPD. We next assessed whether this SNP was associated with clinical phenotype by comparing frequent and infrequent exacerbators (Table 1). Comparing the frequency of the SNP by exacerbation frequency using Fisher's exact test, we found that the rs2227744A allele was less common in the frequent than the infrequent exacerbator phenotype (Table 3). Reflecting this, and considering exacerbations as a continuous variable, the presence of the minor allele of the SNP was associated with a significantly lower exacerbation rate (Mann-Whitney *U*-test, 1.98 vs. 3.03 exacerbations/year, *P* = 0.04). The lack of association of this SNP with the development of COPD was replicated in two additional COPD cohorts identified within the Whitehall II and ELSA participants. The SNP was in Hardy-Weinberg equilibrium in both populations (data not shown). Logistic regression analyses confirmed the lack of association between the SNP and COPD, before (data not shown) and after correction for age and sex (Table 3). Relationship between the SNP genotypes and lung function was investigated in all the cohorts and no significant association was found (Table 4).

**Table 3.** PAR-1 Polymorphisms and susceptibility to COPD and exacerbations

	Cases	Controls	Minor Allele Frequency Cases	Minor Allele Frequency Controls	*P* Value	OR (95% CI)
London cohort	203	609	0.44	0.46	0.34	0.90 (0.71–1.12)
WHII	318	1133	0.48	0.45	0.18	1.13 (0.94–1.36)
ELSA	364	1094	0.47	0.46	0.58	1.05 (0.88–1.25)

	FE	IE	Minor Allele Frequency FE	Minor Allele Frequency IE		
FE vs. IE	67	136	0.37	0.46	0.04*	0.64 (0.42–0.98)

Analysis of rs2227744G>A allele frequency differences in COPD. OR, odds ratio; CI, confidence interval. Significant results are indicated with an asterisk. Differences between cases and controls in the London cohort were performed by means of an allelic χ^2^ test. Differences between cases and controls in the Whitehall II and ELSA cohorts were performed by logistic regression with correction for age and sex. Differences between frequent exacerbators (FE) and infrequent exacerbators (IE) were tested with a Fisher's exact test.

**Table 4.** PAR-1 and lung function in COPD

	GG	GA	AA	*P* Value
London cohort				
FEV_1_, liters	1.10	1.24	1.20	0.25
FEV_1_, % predicted	49	50	50	0.75
FVC, liters	2.32	2.63	2.53	0.19
ELSA cohort				
FEV_1_, liters	1.38	1.37	1.39	0.90
FEV_1_, % predicted	56	56	55	0.81
FVC, liters	2.70	2.59	2.60	0.45
Whitehall II cohort				
FEV_1_, liters	2.15	1.97	2.10	0.60
FEV_1_, % predicted	61	58	61	0.98
FVC, liters	3.67	3.40	3.51	0.25

Analysis of rs2227744G>A and lung function relationship in COPD.

## DISCUSSION

PAR-1 plays a critical role in mediating the interplay between coagulation and inflammation and represents an interesting candidate gene in the setting of COPD, a condition in which both pulmonary and systemic coagulation and inflammation are increased (7, 25). PAR-1 polymorphisms have been investigated in other disease contexts and positive associations have been found for venous thromboembolism (37), coronary heart disease (28), liver fibrosis (24), and MI (15). Interestingly, in the context of heart disease, PAR-1 variants, including the rs2227744A allele located in the *F2R* promoter, have been shown to associate with increased inflammation and MI (15). Despite this, to the best of our knowledge, the function of the rs2227744G>A variant is unknown and its involvement in COPD has not been investigated. The aim of this study was to determine whether this genetic variant may track with COPD and COPD exacerbation frequency, based on our previous data in mice suggesting that *F2R* may affect the development of key COPD phenotypes (3).

Using reporter gene assays and a 5.2-kb promoter construct transfected into HeLa cells, we show that the rs2227744A minor allele increases PAR-1 expression by almost threefold. In silico analysis using common free-access online tools suggests that this SNP is not directly located in a binding site for transcription factors and that the minor allele does not introduce a new transcription factor binding site. However, this SNP is located within a DNAse I hypersensitivity region upstream of the main transcription factor binding cluster for this gene. This raises the possibility that this SNP may promote PAR-1 gene expression by influencing the availability of binding sites at nearby transcription factor binding site locations. Given the role of PAR-1 in influencing multiple downstream inflammatory pathways, the observation that this polymorphism is functional and impacts on PAR-1 gene transcription may explain the previous report that associated the rs2227744A allele with higher inflammation in subjects at risk of MI (15). In contrast, our data show that there is no association between this SNP and the development of COPD in three large Caucasian cohorts. Similarly, the SNP was found not to be associated with lung function in all the cohorts. However, comparing frequent and infrequent exacerbators in our cohort of COPD patients, we found that the minor allele of the SNP associated with fewer exacerbations. To the best of our knowledge, this is the first study to investigate the impact of a functional polymorphism in the PAR-1 promoter on the development of COPD and the determination of COPD phenotypes.

Although we fully acknowledge that our COPD cohort is small, our study was performed on carefully phenotyped patients who were monitored on a daily basis over many years with exacerbations identified by use of previously described and validated criteria of respiratory symptom increase (11). Traditionally, the view has been that frequent COPD exacerbator status may be associated with increased inflammatory responses, and it is known that both IL6 and fibrinogen levels are increased at exacerbation (40). On the basis of our data, it is therefore tempting to speculate that, in COPD, higher levels of PAR-1 gene expression above a certain threshold contributed, at least in part, by the rs2227744A allele might support an appropriate PAR-1-mediated inflammatory response that leads to early resolution of disease and resistance to COPD exacerbation. In contrast, PAR-1 expression levels that fail to lead to an appropriate PAR-1-mediated inflammatory response may support the continuation of chronic inflammation. It is also possible that the absence of an appropriate PAR-1-mediated inflammatory response in response to a stimulus predisposes to recurrent infections that characterize the frequent COPD exacerbator phenotype. Inflammation is particularly important at exacerbation of COPD, when there is also evidence of increased activation of coagulation (40). This may be particularly important in relation to the interplay between COPD, COPD exacerbations, and cardiovascular risk (29).

Studies in murine models focusing on the role of PAR-1 in the context of bacterial infection have revealed that the role of this receptor during infection is highly context and time dependent and both can be advantageous by influencing a robust inflammatory response but also may be deleterious and promote bystander tissue damage (1, 21, 22, 26, 34). This may in part explain the paradoxical role of the SNP in the context of COPD reported in the present article in that the function of the rs2227744 SNP (i.e., higher PAR-1 expression) only manifests itself during episodes of exacerbation since the advantageous proinflammatory response could aid the control of bacterial infection (a major cause of COPD exacerbation).

Several GWAS have now been conducted in COPD and several genes have been found to be associated with the development of this condition, the most common and strongly associated being *HHIP* (hedgehog-interacting protein) (30), *CHRNA3* (cholinergic receptor, nicotinic, alpha 3) (30), *IREB2* (iron responsive element binding protein 2) (10), and *FAM13A* (family with sequence similarity 13, member A) (8). *CHRNA3* has been demonstrated to be associated with smoking intensity (30) and all show an association with FEV_1_/FVC (16, 30) but, to the best of our knowledge, there are no studies investigating associations with exacerbation frequency or other key phenotypes in COPD. In studies like the one reported here, where the frequency of the rs2227744A allele in infrequent exacerbators was found to be similar to that observed in the general population (Whitehall II and ELSA healthy controls), and the effect of the SNP on a subgroup of cases was diluted and masked, a comparison between all COPD cases (including both frequent and infrequent exacerbators) and healthy controls would potentially fail to show an association between PAR-1 and susceptibility to COPD. On the contrary, highly defined cohorts such as ours may have lower statistical power, but the level of phenotypic characterization achieved is higher than that of large cohorts, allowing the investigation of associations with carefully defined subgroups and the detection of the effect of genes with small effects only on a particular phenotype. There is increasing evidence that the frequent exacerbator represents an independent phenotype (18), but despite indication of familial aggregation (14), studies examining genetic determinants of exacerbation frequency remain limited. This reflects a need for the collection of large cohorts with the same degree of careful phenotyping as exemplified by our cohort. It is clear that this will require a major multicenter, collaborative effort with highly engaged patients.

This study has some limitations, due to the degree of characterization required to properly estimate the exacerbation frequency. First, the number of cases for which obtaining the exacerbation frequency was possible is small. This negatively affects the power of the analyses. Second, the analyses regarding the association of the SNP with the exacerbation frequency need replication but this cannot at present be achieved, owing to the lack of a similarly characterized cohort.

## CONCLUSION

In conclusion, although this study demonstrates a lack of correlation between a functional PAR-1 polymorphism and susceptibility to COPD in three different UK cohorts, this is the first report suggesting that the rs2227744A allele is associated with reduced susceptibility to frequent exacerbation. Although the mechanism by which this polymorphism may potentially influence this important clinical phenotype remains at present unknown, we hypothesize that PAR-1 signaling and in particular its role in modulating inflammatory responses may play an important role in the determination of disease phenotype, a little-studied and evolving field for which our report provides a new model. We believe that our data further add patient-based evidence to our current understanding of the potential role of pathological coagulation signaling responses in promoting inflammatory and potentially remodeling pathways in response to chronic lung injury. Future studies aimed at replicating these findings and at further defining the relationship between coagulation signaling events and inflammation in these disease contexts are urgently required and may shed important light on the pathomechanisms underlying the development and progression of these conditions.

## GRANTS

This work was supported by a British Lung Foundation, Garfield Weston Foundation award (Grant reference COPD10-6), and a Wellcome Trust Clinical Training Fellowship (074558). The Whitehall II study has been supported by grants from the Medical Research Council (MRC); Economic and Social Research Council; British Heart Foundation (RG/02/005; PG/03/029); Health and Safety Executive; Department of Health; National Heart Lung and Blood Institute (HL36310); US NIH National Institute on Aging (AG13196); US NIH Agency for Health Care Policy Research (HS06516); and the John D. and Catherine T. MacArthur Foundation Research Networks on Successful Midlife Development and Socioeconomic Status and Health. The US National Institute on Aging (grant numbers R01AG24233, R01AG1764406S1) funds the English Longitudinal Study of Ageing DNA Repository (EDNAR), and ELSA is funded by the National Institute of Aging in the USA and a consortium of UK Government departments coordinated by the Office for National Statistics. The London COPD cohort is funded by the MRC, UK.

## DISCLOSURES

All authors report that no potential conflict of interest exists with any companies/organizations whose products or services may be discussed in this article.

## AUTHOR CONTRIBUTIONS

M.P., P.J.L., and J.K.Q. performed experiments; M.P. analyzed data; M.P., R.C.C., M.R.H., and J.R.H. interpreted results of experiments; M.P. prepared figures; M.P. and J.R.H. drafted manuscript; M.P., R.C.C., and J.R.H. edited and revised manuscript; M.P., P.J.L., M.R.H., J.K.Q., M.K., G.J.L., J.A.W., R.C.C., and J.R.H. approved final version of manuscript; M.R.H., M.K., G.J.L., J.A.W., R.C.C., and J.R.H. conception and design of research.
